# Pulmonary function tests in patients with amyotrophic lateral sclerosis and the association between these tests and survival

**Published:** 2014-07-04

**Authors:** Seyed-Ali Javad Mousavi, Babak Zamani, Shahab Shahabi Shahmiri, Mohammad Rohani, Gholam Ali Shahidi, Elyas Mostafapour, Helia Hemasian, Hanieh Raji

**Affiliations:** 1 Department of Internal Medicine, School of Medicine, Iran University of Medical Sciences, Tehran, Iran; 2 Department of Neurology, School of Medicine, Iran University of Medical Sciences, Tehran, Iran; 3 Department of Scientific Research Center, School of Medicine, Iran University of Medical Sciences, Tehran, Iran; 4 Department of Neurology, School of Medicine, Isfahan University of Medical Sciences, Isfahan, Iran; 5 Department of Internal Medicine, School of Medicine, Ahvaz Jundishapur University of Medical Sciences, Ahvaz, Iran

**Keywords:** Amyotrophic Lateral Sclerosis, Pulmonary Function Tests, Survival

## Abstract

**Background: **The rapidity of progression of amyotrophic lateral sclerosis (ALS) to death or respiratory failure impacts patients, clinicians, and clinical investigators. The aim of this study is to evaluate of the pulmonary function tests (PFTs) in patients with ALS and the association between these PFTs and survival

**Methods:** A total of 36 ALS patients who PFTs, including vital capacity (VC), maximum mid-expiratory flow rate (MMEFR), forced vital capacity (FVC), and forced expiratory volume in 1 s (FEV_1_), were available from the time of diagnosis were included in this study. Non-pulmonary characteristics assessed at the time of PFTs. Data were analyzed using chi-square, Student’s independent t-test, Kaplan-Meier, correlation, and receiver operating characteristic (ROC) curve.

**Results: **The mean age of subjects was 55.36 (SD = 12.24) year, and the male to female ratio was 2.6. Twenty-five (69.4%) were died in 5 years period of our study. The mean and median survival time (In months) was calculated as 42.51 (95% confidence interval [CI] 33.64-51.39) and 38 (95% CI 27.23-48.77) months, respectively. The rate of ALS survival was 74% at 1^st^ year, 41% at 3^rd^ year and 10% at 5^th^ year of starting symptoms. The results of Kaplan-Meier test showed survival was significantly longer in the group with PFTs closer to normal. In addition, ROC analysis showed that FVC < 50% could potentially be a predictor of death in ALS patients(P = 0.003, area under curve = 0.649).

**Conclusion: **We found single measures of upright FVC, FEV_1_ to be significantly associated with survival, even after controlling for relevant non-pulmonary patient characteristics. Our study demonstrated that upright FVC, FEV_1_, VC, and MMEFR are useful non-invasive measures in the prediction of survival in ALS.

## Introduction

Amyotrophic lateral sclerosis (ALS) is a progressive neurodegenerative disease that ultimately leads to respiratory failure and death. ALS is the most common form of the motor neuron diseases. A large degree of inter-patient variability exists in the rate of progression, with some patients die or require respiratory support within months and others have relatively prolonged survival.^1^^-^^4^ The consequent uncertainty in survival is not only difficult for patients and their families, but also frustrates research on ALS, as it may be difficult to assemble a cohort of patients that can be expected to complete a clinical trial. Certain non-pulmonary factors, including female gender, advanced age, short time from symptoms onset to diagnosis, and bulbar onset of disease are associated with shorter survival.^5^^-^^7^ One clinically relevant prognostic factor in patients with ALS is the time of onset of respiratory muscle weakness. Patients with respiratory muscle weakness as the initial manifestation of ALS have very poor prognosis, with a median survival from diagnosis of only 2 months. It is crucial, therefore, to accurately detect respiratory muscle involvement in order to estimate prognosis, provide patient counseling, and make treatment decisions. Most people with ALS die from respiratory failure, usually within 3-5 years from the onset of symptoms. However, as ALS overwhelmingly brings death through respiratory compromise, pulmonary function tests (PFTs) should be particularly appropriate predictors of individual survival.^8^^,^^9^ The aim of this study is to evaluate of the PFTs in patients with ALS and the association between these PFTs and survival.

## Materials and Methods

Records were reviewed of all ALS patients who were referred to neurology clinic of Rasoul-e-Akram Hospital in Tehran, Iran for the period 2006-2011. Those patients who fulfilled El Escorial criteria for “definite” or “probable” ALS and in whom PFTs, including vital capacity (VC), maximum mid-expiratory flow rate (MMEFR), forced vital capacity (FVC), and forced expiratory volume in 1 s (FEV_1_), were available from the time of diagnosis were included in this study.

Patients were excluded from analysis if they had a prior history of symptomatic pulmonary disease unrelated to ALS. Our Institutional Committee on Clinical Investigation approved the study protocol.

***Independent variables***

Non-pulmonary characteristics assessed at the time of PFTs including gender, age, clinical area of disease onset (Bulbar vs. extremities), time from symptoms onset to diagnosis, time from symptoms onset to spirometry, arterial blood gas (ABG) findings (P_a_CO_2_, P_a_O_2_, and HCO_3_), ALS Functional Rating Scale (ALSFRS) scores at the first and last visit, electromyography finding (distal latency of ulnar and median sensory and motor nerves, compound motor action potentials, and motor amplitudes), laboratory findings (Alanine transaminase, aspartate transaminase, and creatine phosphokinase) and neurologic finding (Fasciculation, jaw jerk, and deep tendon reflexes). The prescription of riluzole (Rilutek; Aventis, Strasbourg, France) or non-invasive positive pressure ventilation (NPPV) during the period of observation was recorded.

***PFT***

All tests were performed in the Rasoul-e-Akram Hospital pulmonary function laboratory. Spirometry was performed in the upright-seated position. FVC, FEV_1_, MMEFR, and vital capacity (VC) were measured on a spirometer. Using the formulae of Goldman and Becklake, predicted values were determined. These standards were used to determine percent predicted upright FVC, FEV_1_, VC, and MMEFR.

***ALSFRS***

The ALSFRS^3^^,^^10^ is a 10-item functional inventory which was devised for use in therapeutic trials in ALS. Each item is rated on a 0 (unable) to 4 (normal) scale by the patient and/or caregiver, yielding a maximum score of 40 points. The ALSFRS assesses patients’ levels of self-sufficiency in areas of feeding, grooming, ambulation, and communication.

***Outcome measurements***

The patient’s survival was obtained through telephone interviews with each patient or the patient’s surviving family. If contact was unobtainable, the outcome was determined through hospital records.

***Statistical analyses***

Data were analyzed using SPSS for Windows 18.0 (SPSS Inc., Chicago, IL, USA). All data are expressed as mean ± SD. The distribution of nominal variables was compared using the chi-squared test. In order to compare the mean values of quantitative variables the Student’s independent t-test and one-way ANOVA procedures were performed. Survival was calculated from the time of the patients’ spirometry testing. Survival rate was carried out using the methods of Kaplan-Meier. To adjust for potential confounding by other factors historically associated with survival, Cox proportional hazard modeling was used for the multivariate analysis of each pulmonary predictor with age, area of disease onset, time from symptom onset to diagnosis, and riluzole usage.

After the determination of the significance of each test in predicting death, attention was shifted to the tests’ utility in predicting this outcome at 1 year of follow-up, a time point applicable to many clinical trials. Sensitivity, specificity, positive and negative predictive value of each variable was determined with regard to predicting death at that time. Clinically relevant cut-points for supine and upright FVC were severe (<50% predicted FVC), moderate (50-69%), mild (69-79%), and no (>80%) restriction. Receiver operating characteristic (ROC) curves, for example, plots of sensitivity/ (1 - specificity) at each potential test cut-point was determined. The area under the ROC curve, a measurement representative of the overall desirability of a diagnostic test, was determined for each pulmonary test. Statistical tests were performed to compare the areas under the tests’ curves.

## Results

A total number of 36 patients were included in our study with the mean age of 55.36 (SD = 12.24) years ranged between 28 and 81 years old. The patients were 26 (72.2%) male and 10 (27.8%) female.

During the period of observation, riluzole was prescribed for 27 (75%) of the patients; NPPV support such as bi-level positive airway pressure (BiPAP) ventilation was prescribed for 21%. Seven (19.4%), 20 (55.6%), and 9 (25%) had upper limb, lower limb, and bulbar onset of disease, respectively. The mean time from symptom onset to ALS diagnosis was 16.27 (SD = 7.33) months. Spirometry was performed a mean of 19.38 (SD = 9.36) months after symptom onset. The mean upright FVC was 64.32% (SD = 28.74%) of predicted, corresponding to a mild to moderate restrictive deficit. The mean of ALSFRS of patients at the first visit was 27.69 (SD = 7.33) ranged 9-39. This amount was changed to 20.44 (SD = 8.06) at the last visit. Although the mean PaCO_2_ was normal 40.02 (SD = 7.96), there was a wide range (27-60 mm Hg), with some patients displaying hypercapneic ventilatory failure at the time of baseline spirometry. In neurologic examination; upper limb, lower limb reflexes and jaw jerk were increased in 71.4%, 71.4%, and 41.4%, respectively. Tongue, trunk, upper and lower limb fasciculation were seen in 58.6%, 42.9%, 79.3%, and 65.4%, respectively. None of the patients had any family history of ALS.

The differences of the characteristics between patients with less or more than 5 years survival are summarized in table 1. As it is shown, the mean value percent predicted of FVC was 54.68 (SD = 24.72) and 86.24 (SD = 25.75), in dead and alive patients, respectively. Student’s independent t-test analysis demonstrated that this difference was statistically significant (P = 0.004).

**Table 1:** Demographic and clinical main variables of the amyotrophic lateral sclerosis patients with less or more than 5 years survival

**Variables**	**Less than 5 years survival (n = 25)**	**More than 5 years survival (n = 11)**	**P**
Age at ALS onset (years), mean (± SD)	58.23 (11.83)	48.83 (11.18)	0.033
Gender			
Female	7 (28)	3 (27)	0.960
Male	18 (72)	8 (73)	
FVC, % predicted, mean (± SD)	54.68 (24.72)	86.24 (25.75)	0.004
FEV_1_, % predicted, mean (± SD)	60.12 (28.89)	91.20 (25.58)	0.001
PaCO_2_ mmHg, mean (± SD)	40.90 (8.96)	37.83 (4.28)	0.180
Riluzole treatment, n (%)	18 (72)	9 (81)	0.530
ALSFRS at first visit, mean (± SD)	26.88 (7.77)	29.54 (6.15)	0.280
ALSFRS at last visit, mean (± SD)	18.63 (8.48)	24.75 (5.14)	0.032
ALS onset			
Upper limb, n (%)	3 (12.0)	4 (36.5)	0.233
Lower limb, n (%)	15 (60.0)	5 (45.5)	
Bulbar, n (%)	7 (28)	2 (18)	

ALS: Amyotrophic lateral sclerosis; SD: Standard deviation; FVC: Forced vital capacity; FEV_1_: Forced expiratory volume in 1 s

ALSFRS: Amyotrophic lateral sclerosis functional rating scale

**Figure 1:**
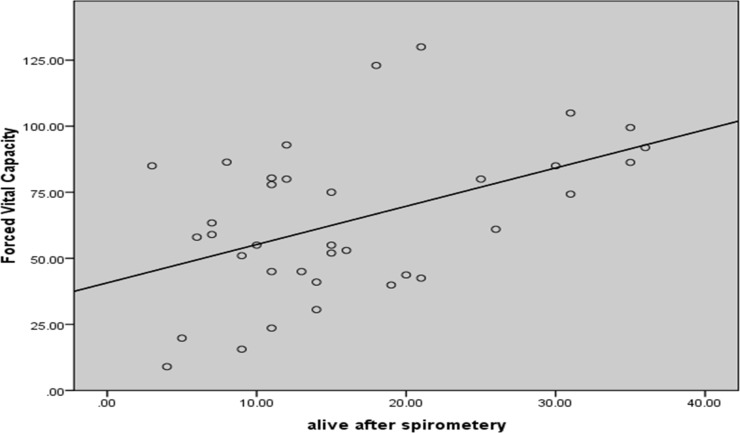
Scatter plot of correlations between percent predicted of forced vital capacity and patient’s survival after spirometery (In month)

Evaluation of FVC status qualitatively (Severe, moderate, mild, and no restriction) with chi-square analysis showed that severe restriction (<50% predicted FVC), was significantly more common in dead patients (40% vs. 9%, P < 0.001).

There was a significant positive correlation between percent predicted of FVC and patient’s survival after spirometry (In month) (P = 0.004, R_Spearman_ = 0.472) (Figure 1).

***Outcomes and survival analysis***

**Figure 2:**
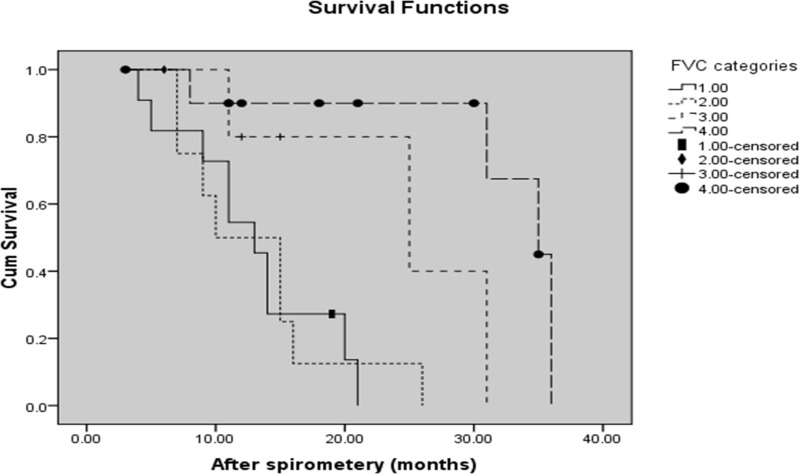
Kaplan-Meier curves showing differences in survival based on values of upright forced vital capacity (FVC). The categories of FVC are as follows: FVC < 50% (line 1); FVC = 51-69% (line 2); FVC = 69-80%  (line 3); FVC > 80% (line 4)

Twenty-five (69.4%) patients had <5 years survival in our study. The mean and median survival time (In months) was calculated as 42.51 (95% confidence interval [CI]: 33.64-51.39) and 38 (95% CI: 27.23- 48.77) months, respectively. The rate of ALS survival was 74% at 1^st^ year, 41% at 3^rd^ year, and 10% at 5^th^ year of disease. The results of Kaplan-Meier test showed survival was significantly longer in the group with values closer to normal (Figure 2).

For non-pulmonary factors (Age, area of onset, time to diagnosis, and riluzole usage), more multivariate analysis showed that the effect of FEV_1_ and FVC qualitative status on the patients’ survival was independent from age, area of onset, time to diagnosis, and riluzole usage (P < 0.05).

**Figure 3:**
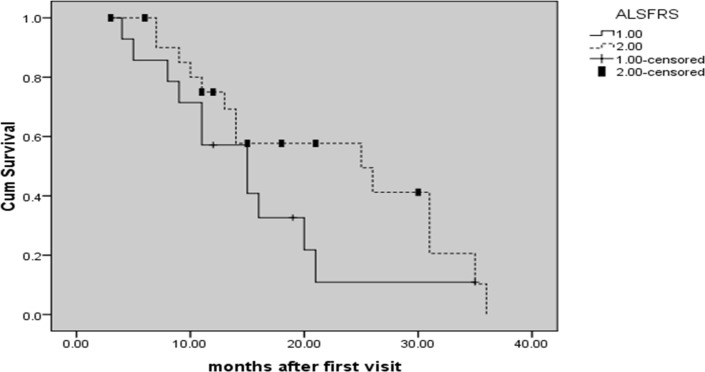
Kaplan-Meier curves showing differences in survival based on values of amyotrophic lateral sclerosis functional rating scale (ALSFRS) score.  The categories ALSFRS score are as follows: ALSFRS score < 27 (line 1); ALSFRS score > 27 (line 2)

Among 36 patients, 14 (32%) had a baseline ALSFRS score of <27 (The median baseline score of all patients). Patients with a total ALSFRS score below the median had increased risk of death median (Mean survival time in months: 15.45, 95% CI: 10.64-20.26) compared to those who scored above the median (Mean survival time in months: 22.47, 95% CI: 17.37-27.56) (P < 0.001) (Figure 3).

**Figure 4:**
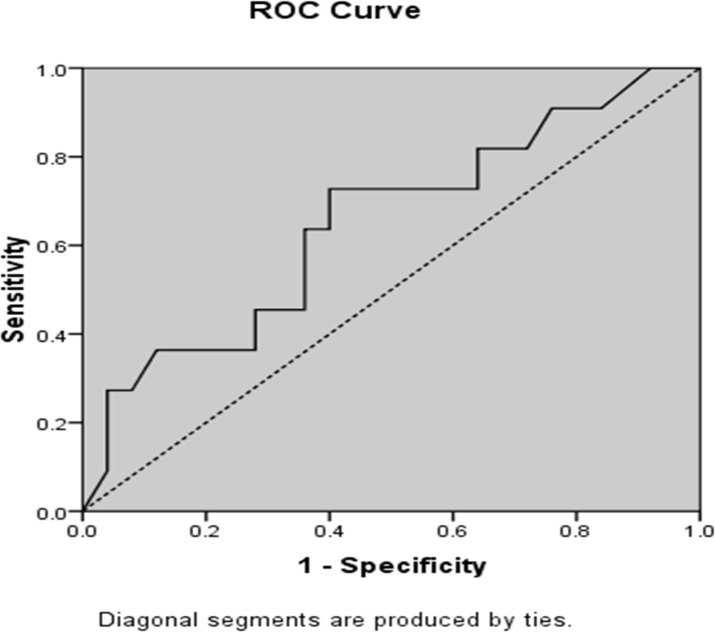
Receiver operating characteristic curve of forced vital capacity < 50% to predict death in amyotrophic lateral sclerosis patients (P = 0.003, area under curve = 0.649)

In addition, ROC analysis showed that FVC < 50% could potentially be a predictor of death in ALS patients (P = 0.003, area under curve = 0.649), results of ROC curve analysis are shown in figure 4.

## Discussion

PFTs are commonly used as a measure of progression in ALS. This study assessed the ability of PFTs to predict survival in ALS patients. The demographic characteristics of our sample are similar to other groups of ALS patients. But we found few differences. For example, results of this study showed that most of the patients were male, and M:F ratio was 2.6. The higher prevalence and incidence of ALS in men have been observed in many other epidemiological studies. However, this ratio among Iranian patients is somewhat more than similar studies. Investigation about the cause of this observation warrant further study.

We found single measures of upright FVC, FEV_1_ to be significantly associated with survival, even after controlling for relevant non-pulmonary patient characteristics. Our study demonstrated that upright FVC, FEV_1_, VC, and MMEFR are useful noninvasive measures in the prediction of survival in ALS.

A patient with a normal FEV_1_ has a >95% chance of survival at 1 year; a normal upright FVC predicts at least a 1-year survival in over 89% of cases. We believe that these findings are important and valuable for ALS care, both in a clinical trial enrollment and inpatient counseling. Normal values were seen in 11 of 36 patients on FVC (30%) and 16 of 36 patients on FEV_1_ (44%). Among the patients, 44% had FVC >65% predicted, and these patients can be expected to have an 86% 1-year survival.

Nakano et al.^11^ were among the first to report serial pulmonary function studies in ALS. They measured lung volume, spirometry, and diffusing capacity, but not static pressure, in 25 patients at beginning 9 months after onset and at 15, 22, and 33 months. Initial mean VC was within the normal and averaged 58 ± 26% of predicted at final measurement.

Fallat et al.^12^ reported spirometry (But not static pressure) at the time of diagnosis and serially in a large number of patients with motor neuron disease, most with ALS. At the time of diagnosis, 93.6% of their patients showed at least one abnormality of the following three parameters: FVC, maximum voluntary ventilation (MMV), and residual volume. Of 45 patients with reduction in FVC and/or MMV of more than 50%, 29 had been clinically judged to have no breathing abnormality, and 15 were thought to be mildly impaired, demonstrating the need for an objective assessment of respiratory muscle strength.

Similar to results of study by Kaufmann and colleague,^13^ we found ALSFRS score at baseline and after adjustment for age, sex, and symptom duration is a strong predictor of death. Ringel et al.^14^ reported the median survival was 4.0 years for the study cohort, but 2.1 years for newly diagnosed cases. They concluded decline in pulmonary function most closely correlated with death. To compare we calculated median survival time 38 months. Rate of decline in PFTs was not measured in our study. Our results focused the importance of considering baseline PFTs in ALS patients.

More recently, Carratu et al.^15^ observed Increased survival rate at 1 year in patients with FVC < 75% treated with NPPV, as compared to those who refused or could not tolerate NPPV (P = 0.020). The median rate of decline in FVC% was slower in NPPV patients than in patients who did not use NPPV (95% CI: 0.72-1.85; P < 0.001).

Several study had one or more measurements of seated and supine PFTs. For example, in 2010 Baumann et al.^7^ observed subjects with abnormal values of seated FVC, supine FVC, maximum inspiratory pressure (MIP) and maximum expiratory pressure had significantly reduced survival compared to subjects with normal values. A normal supine FVC was highly predictive for 2-year survival and had superior sensitivity over seated FVC. Slower rates of decline in seated or supine FVC were strong predictors of 2-year survival. It was similar to study of Lechtzin et al.^16^ Their study showed that supine FVC is an accurate, noninvasive means to estimate Pdi-sniff (Trans-diaphragmatic pressure during maximal sniffing). Combining two tests, supine FVC and MIP, is better than supine FVC alone. Their data showed that supine FVC at <75% predicted is very sensitive and specific for identifying diaphragmatic weakness. Furthermore, in 2006 Schmidt et al.^17^ examined FVC in the supine position, a test known to be a better predictor of diaphragmatic weakness than upright FVC. An abnormal supine FVC is 95% sensitive for death or tracheostomy at 1 year; conversely, a normal supine FVC has an 83% predictive value for survival at this time. A normal upright FVC only has a 70% predictive value.

The results of Schiffman and Belsh’s study^18^ showed, VC (Percent predicted) was significantly lower in the symptomatic group (55.9 ± 20.3) compared with the asymptomatic group (76.4 ± 21.0). Respiratory muscle impairment as measured by VC (Percent predicted) was related to stage of disease at presentation. Rate of decline of respiratory muscle strength as measured by VC (−3.5%/month), negative inspiratory pressure (+2.9 cm H_2_O/month), and positive expiratory pressure (−3.4 cm H_2_O/month) tended to be linear with a great deal of inter-patient variability.

In a placebo-controlled trial of riluzole treatment in 959 patients with ALS by Riviere,^19^ their results showed a significant difference in time to transit between the riluzole and the placebo groups in milder states of ALS. However, riluzole therapy did not demonstrate any benefit in patients with more advanced ALS. The studies by Kleopa et al.^20^ and Aboussouan et al.^21^ agree in their observations of poorer survival times in patients with bulbar involvement who did not tolerate non-invasive ventilation (NIV). Recently, Weikamp et al.^22^ showed tongue strength is an independent prognostic factor for survival time in patients with ALS.

However, we did not observe differences in the survival times of patients with bulbar involvement or riluzole administration in our study.

Additional measurements of pulmonary function are pulse oximetry and ABG levels. Although the mean PaCO_2_ was normal 40.02 (7.96), there was a wide range (27-60 mm Hg) in PaCO_2_, some patients displayed hypercapneic ventilatory failure at the time of baseline spirometry. Reason for these normal ABG levels was carbon dioxide rises late in the course of ALS.

Our study has certain limitations. We were not able to quantify patient adherence with NIV and other potential confounders of survival. The majority of subjects were (At some point after study enrollment) prescribed NIV.

Any confounding influence, however, is limited by our finding that BiPAP use did not change the predictive value of upright FVC, FEV_1_, and VC, each of which remained statistically significant regardless of patients’ eventual use of NIV. This finding does not contradict the wealth of previous literature supporting BiPAP use (or our own opinions regarding BiPAP’s usefulness in the treatment of patients with ALS). Because of limitations of our study design with respect to BiPAP use (Indeed, NIV use was more common in subjects with abnormal PFTs), our study should not be used to draw conclusions about this intervention’s utility in the care of the ALS patient. Another limitation in our study was some patients did not cooperate in spirometry, and some of them excluded because they did not follow up. For this reason, we had limitation in serial spirometry and in the supine position.

## Conclusion

ALS is a devastating disease that invariably leads to respiratory failure. Abnormal spirometric variables such as the FVC and FEV_1_, likely reflect respiratory muscle weakness. Serial PFTs are an important part of the management of a patient with ALS. Soon after diagnosis, a baseline PFTs should be performed. The tests are an objective measure of respiratory muscle function, and because measurements are made on a linear scale, are helpful in judging the overall progression of the disease.

## Notes

**How to cite this article: **Mousavi SAJ, Zamani B, Shahabi Shahmiri Sh, Rohani M, Shahidi GhA, Mostafapour E, et al. Pulmonary function tests in patients with amyotrophic lateral sclerosis and the association between these tests and survival. Iran J Neurol 2014; 13(3): 131-7.
